# Effectiveness of online asthma training programmes to improve asthma management among school staff: a systematic review using the Kirkpatrick evaluation model

**DOI:** 10.1038/s41533-025-00450-w

**Published:** 2025-10-23

**Authors:** Muhammad Alieff Isqandar Jefnee, Munita Kaur, Chin Hai Teo, Sheron Sir Loon Goh, Pauline Siew Mei Lai, Christine Shamala Selvaraj, Siti Nurkamilla Ramdzan

**Affiliations:** 1 Department of Primary Care Medicine, Faculty of Medicine, Universiti Malaya, 50603 Kuala Lumpur, Malaysia; 2 Department of Clinical Pharmacy and Pharmacy Practice, Faculty of Pharmacy, Universiti Malaya, 50603 Kuala Lumpur, Malaysia; 3 Sir Jeffrey Cheah Sunway Medical School, Faculty of Medical and Life Sciences, Sunway University, 47500 Sunway City, Malaysia

**Keywords:** Diseases, Health care, Medical research

## Abstract

Asthma is a leading chronic illness in children worldwide, and school staff are often the first responders when asthma emergencies occur at school. Despite their crucial role, many school staff lack adequate training in asthma management. Online training has emerged as a standardised and scalable solution, but its broader effectiveness remains uncertain. This systematic review aimed to evaluate the effectiveness of online asthma training programmes for school staff. A comprehensive search was conducted across six databases (PubMed, CINAHL, Scopus, Web of Science, ProQuest, and Education Research Complete) in June 2024. Eligible studies included those that evaluated online asthma training programmes targeting school staff (teachers, classroom assistants, and school nurses). The effectiveness of interventions was assessed using the Kirkpatrick evaluation model, which categorises outcomes into four levels: reaction, learning, behaviour, and results. Methodological quality was appraised using the Mixed Methods Appraisal Tool (MMAT). Eight studies met the inclusion criteria with quality scores ranging from 40 to 80%. Interventions identified included web-based modules (*n* = 4), online classrooms (*n* = 2), an eBook (*n* = 1), and a PowerPoint presentation (*n* = 1). All reported participants’ positive satisfaction with the training and improvements in asthma knowledge (levels 1 and 2). However, none of the studies evaluated behavioural change or organisational outcomes (levels 3 and 4). Most studies had small sample sizes and lacked long-term follow-up, limiting assessment of real-world impact. Online asthma training programmes improve school staff’s knowledge and satisfaction, and appear to be as effective as face-to-face training. However, their impact on behavioural or organisational change remains lacking. Future research should explore long-term effects to support real-world implementation.

## Introduction

Asthma is the most common chronic disease affecting children worldwide, with a global prevalence of approximately 10%^1–3^. Children with asthma, especially those below 10 years old, often rely on adults for support, as they may lack the capacity to independently manage their asthma^4,5^. Children depend on parents and teachers to assist them at home and in school, respectively. However, schools in low- and middle-income countries (LMICs) lack in supporting asthma self-management due to the absence of a school asthma guideline and training^6,7^. The World Health Organization (WHO) recommended that school staff support the self-management of children with asthma and receive training to act as first-line responders during asthma emergencies^8–10^.

Studies have highlighted gaps in school staff’s knowledge and preparedness in managing asthma, leading to delays in treatment and increased risk during emergencies^6,7,11,12^. Several studies have shown that school staff often lack adequate training in asthma management and may hold misconceptions about asthma symptoms, medication use, and triggers^5,9,13,14^. In response, various asthma education interventions, including pamphlets, personalised action plans, structured training, and pharmacist-led sessions were conducted and proven effective in improving school staff’s knowledge and confidence across different countries, including Malaysia^15–21^. However, widespread implementation of face-to-face training remains challenging due to logistical and financial constraints, especially in rural or under-resourced settings^22^. Online training has emerged as a scalable and accessible alternative, but evidence of its effectiveness remains limited. Therefore, this systematic review aims to assess the effectiveness of online asthma training programmes for school staff.

## Methods

This systematic review was registered on PROSPERO (registration number: CRD42024562546) and conducted following the Preferred Reporting Items for Systematic Reviews and Meta-Analyses (PRISMA) guidelines^23^.

### Study eligibility criteria

All randomised controlled trials (RCTs), non-randomised controlled studies, and pre-post studies were included if they assessed the effectiveness of online asthma training programmes. These study designs allow for before-and-after comparisons, which are essential for assessing the effectiveness of training programmes. RCTs and non-randomised studies can help control for confounding variables, while pre-post designs provide baseline and follow-up data to measure learning outcomes. Studies were included if the online asthma training programmes were developed for teachers, final-year Bachelor of Education students, classroom assistants (staff who support teachers with classroom activities), and school nurses. In some systems, school nurses are responsible for student health, whereas in others, this role is undertaken by a designated teacher or classroom assistant. Therefore, we consider these groups collectively as school staff. Training programmes could be in any format, such as webinars, training courses, live virtual classrooms, or eBooks. To assess the effectiveness of the programmes, studies must measure asthma knowledge before and after the training as one of the study outcomes. Original studies published in English and available as full-text were included. Articles were excluded if they were published solely as editorials, commentaries, brief reports, expert opinions, case studies, or conference abstracts.

### Information sources and search strategy

A literature search was performed in June 2024 across six databases: PubMed, CINAHL, Scopus, Web of Science, ProQuest, and Education Research Complete using a search strategy based on the PICO (Population, Intervention, Comparison, Outcome) method^24^. Keywords used were “asthma”, “technology”, “school”, and “teacher”. A combination of Medical Subject Headings (MeSH) and free text terms was used to define the search terms. The full search strategy is added as Supplementary Material 1. Reference mining was performed (via forward and backward citation tracking) to identify additional relevant studies. Additionally, nine experts were contacted for study recommendations: eight were corresponding authors of the included studies, and one was a paediatric asthma expert.

### Study selection

Results of the search strategies were combined to yield a pool of preliminary studies. All studies downloaded from the databases were uploaded to Rayyan AI (https://www.rayyan.ai/) (Massachusetts, United States of America), a web-based software designed to assist with conducting systematic reviews^25^. Duplicate studies with the same title and author were excluded via EndNote (version 21) and Rayyan AI. Titles and abstracts were reviewed independently by authors MAIJ and MK for relevance. Full texts of any relevant titles/abstracts were retrieved and assessed for inclusion. Any discrepancies were resolved through discussion between the two reviewers or by consulting a third reviewer, CHT.

### Quality assessment

The quality of each study was assessed independently by authors MAIJ and MK using the Mixed Methods Appraisal Tool (MMAT, version 2018)^26^. MMAT allows for the assessment of different study designs, including qualitative, quantitative, and mixed methods studies. It consists of five criteria that address specific methodological concerns specific to the study design. For clarity and ease of interpretation, we chose to present the MMAT scores for the included studies, ranging from 0% (none of the quality criteria are met) to 100% (all quality criteria are met)^27^. All corresponding authors were contacted to clarify items rated as “Can’t tell” in the MMAT. In cases where clarification could not be obtained, scoring was based on the best interpretation of the available information.

### Data extraction

Data was extracted and recorded independently by authors MAIJ and MK in a standardised extraction form. When disagreements occurred between the two reviewers, they met to discuss the issues that were raised. After reviewing the evidence, the two reviewers then reached a consensus via discussion or were resolved by consulting a third reviewer, CHT. The included articles were categorised into different levels using the Kirkpatrick evaluation model, facilitating the analysis and presentation of findings^28,29^. The four levels are: (1) level 1 (reaction): assessment of participants’ reactions to the training or learning experience; (2) level 2 (learning): change in participants’ knowledge or skills; (3) level 3 (behaviour): change in participants’ behaviour; (4) level 4 (results): change in organisational practice. All corresponding authors were contacted to obtain any missing or incomplete data.

### Data synthesis

Extracted data were synthesised using the Kirkpatrick evaluation model, which offers a structured approach to assess both short- and long-term impacts of training interventions and enables consistent comparisons across diverse study designs^29^. A narrative synthesis was conducted to identify key patterns in training effectiveness^30^. Effect size was assessed using Cohen’s *d*. Outcome measures were presented as mean and standard deviation (SD). A Cohen’s *d* of <0.2 indicates a small effect, 0.2–0.6 is considered a medium effect, and >0.6 suggests a large effect^31^.

## Results

### Study selection

**Fig. 1:**
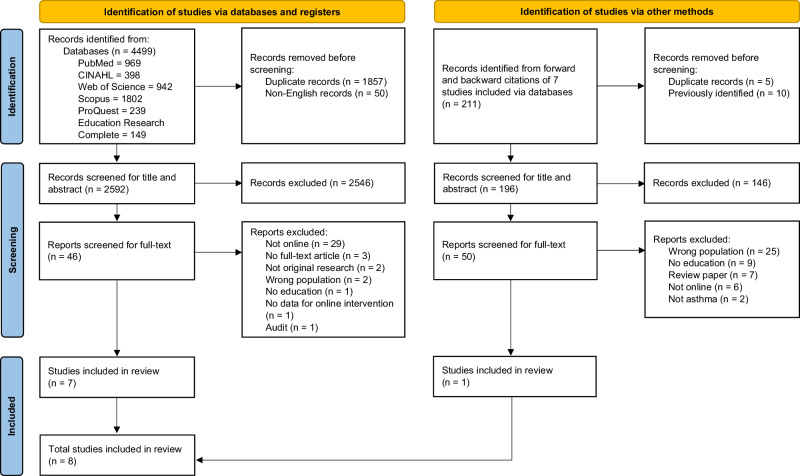
PRISMA diagram for identification and selection of studies.

Eight studies were included in this systematic review (Fig. 1).

### General characteristics of included studies

The systematic review included eight studies conducted across two countries – two in Australia^32,33^ and six in the United States of America (USA)^34–39^. The studies employed different designs, primarily six pre-post studies^33–38^, one RCT^32^, and one non-randomised controlled study^39^. Sample sizes ranged from 10 to 369 participants. Participants were schoolteachers^32,36,37^, school nurses^32,34,35,38,39^, classroom assistants^32^, final-year Bachelor of Education students^33^, and administration staff^32^ (Table 1).

**Table 1:** General characteristics of the included studies.

Study (country)	Study design	Sample size (N)	Target population (*n*)	Follow-up duration for post-test	Type of intervention	Content of the intervention	Questionnaire(s) used	Kirkpatrick level
Francis et al.^32^ (New South Wales, Australia)	Randomised controlled trial (RCT)	eBook training (58)	Teacher (41) Nurse/first-aid (4) Administration (8) Classroom assistant (5)	3 weeks following downloading of eBook	^a^Asthma First Aid Management in Schools training eBook was developed based on the School Champion Asthma Management Program (SCAMP) training material and takes around 60 min to complete.	Information on asthma, triggers, medications, interactive quizzes, picture galleries, videos depicting real-life asthma first aid scenarios, and demonstrations on how to use inhalation devices.	1. Asthma First Aid Knowledge Questionnaire (AFAKQ) 2. Asthma management self-confidence questionnaire (4-item 10-point Likert scale)	2
		Face-to-face training (62)	Teacher (33) Nurse/first-aid (7) Administration (13) Classroom assistant (9)	Right after the training session	Asthma management training through one of SCAMP three-hour metro Sydney face-to-face training sessions delivered by Aiming for Asthma Improvement in Children (AAIC) clinical nurse consultants.	Information on asthma, triggers, medications, and how to use inhalation devices.		
Francisco et al.^34^ (Missouri, USA)	Pre-post study	Online training (42)	School nurses	Right after the training session	Teaming Up for Asthma Control (TUAC), a web-based training programme including a mix of streaming video, interactive media, and print content. The TUAC module is now known as ‘Supporting Asthma Control in the School Setting’ Accessible via: https://umsystem-muextension.catalog.instructure.com/browse/continuing-med-ed/courses/2024-2026-key-concepts-for-rural-practitioners	EPR-3 recommendations: 1) use of objective measures of airflow, 2) coaching for optimal inhalation technique, 3) appropriate use of ICS, and 4) trigger reduction. Video 1: Curriculum Intro Video 2: Student Asthma Literacy Video 3: Expert Asthma Guidelines Video 4: Asthma Care & Management Video 5: Asthma Tools	1. TUAC pre-test (12 items) 2. TUAC post-test (30 items)	2
		Instructor-led (12)		Not reported	In-person assessment of competency			
Hacker-Taylor^35^, (Kentucky, USA)	Pre-post study	12	Kentucky school nurses who work with students aged 11 to 16 years old	Right after completing the American Lung Association’s (ALA) Kickin’ Asthma programme (given 2 weeks to complete)	Kickin’ Asthma training online through the ALA website. Accessible via: https://lung.training/courses/kickin-asthma.html (payment required)	Asthma modules are as follows: Module 1 introduces asthma basics; Module 2 provides education on asthma triggers and prevention; Module 3 provides information on medications and devices to improve outcomes; Module 4 synthesises all information to prepare participants to recognise emergency signs, to self-advocate, and to problem solve.	1. Knowledge and confidence surveys (self-developed) – 12 items (5-point Likert scale)	1,2
Luckie et al.^33^ (Sydney, Australia)	Pre–post study	78	Final year Bachelor of Education (primary school) students from the University of Sydney	3 weeks after the training session	A 60-min single online asthma management education session hosted by Asthma Australia - A series of modules that included voiced-over slide sets and videos. Accessible via: https://asthmaaustralia.thinkific.com/bundles/asthma-first-aid-for-schools	Asthma facts, asthma management and asthma medications, as well as the management of an acute asthma exacerbation using asthma first aid (AFA). Module 1: Asthma First Aid Module 2: About Asthma Module 3: Asthma at School	1. Asthma First Aid Knowledge Questionnaire (AFAKQ) 2. Scenario-based skills assessment checklist (13-item)	2
McAuliff^36^ (Oklahoma, USA)	Pre-post study	10	Staff and faculty members at one of City of Jenks Elementary School	After reviewing the asthma education presentation	^a^PowerPoint presentation	Asthma diagnosis, asthma triggers, signs and symptoms of asthma exacerbation, asthma treatment, and when to seek help.	1. Newcastle Asthma Knowledge Questionnaire (NAKQ)	2
Nowakowski et al.^37^ (Florida, USA)	Pre-post study	Face-to-face (2011–2012 programme year) (203)	Education professionals employed at public schools throughout Florida	No post-test for knowledge	1-h Asthma 101 face-to-face training	Basic asthma anatomy and physiology, signs and symptoms of respiratory distress, when and how to give medications, Asthma Action Plans (AAPs), how to use asthma management equipment, how to make environmental modifications to control triggers, and other related content.	1. Standard pre- and post-tests for knowledge assessment provided by the ALA. (11 items) 2. Satisfaction survey (6 items)	1,2
		Face-to-face (2012–2013 programme year) (562)		Right after the training session				
		Online (2013–2014 programme year) (369)		Immediately after taking the course & 2 months after taking the course	^a^1-h Asthma 101 course delivered through the Florida Training Finder Real-time Affiliate Integrated Network (TRAIN) course management system, utilising the same slides that were used in face-to-face training. Update by author: Asthma 101 has been officially retired and replaced with Asthma Basics, developed by ALA. Accessible via: https://lung.training/courses/asthma_basics.html (requires formal approval by ALA)			
Nwabuzor^38^ (Western region of USA)	Pre-post study	14	School nurses from a large public school district in the western region of the United States	2 weeks after the asthma educational programme	^a^Asthma Education for School Nurses was delivered virtually using Google Classroom (1-h evidence-based asthma educational programme).	Definition of asthma, negative consequences of asthma, myths and facts about asthma, causes of asthma in children, classification of asthma, diagnosing asthma in children, symptoms of asthma, asthma and exercise, asthma and COVID–19, asthma triggers, asthma management, the effect of asthma on student learning, and how to manage an asthma episode at school.	1. Asthma Knowledge Survey for School Nurses (24 items) 2. Asthma Self-efficacy Survey for School Nurses – 19 items (4-point Likert scale)	2
Putman-Casdorph & Pinto^39^ (West Virginia)	Non-randomised controlled study	Synchronous group (6)	School nurses (North Central West Virginia public schools)	Right after the training session	^a^Wimba live classroom (a web-based conference software) - Asthma module in a live format with the instructor present online at a distant site teaching the asthma module.	Asthma education module including basic asthma information following the NIH, NAEPP Asthma Care Guidelines (2007). The content included information about the pathophysiology of asthma, medications, and devices used to treat asthma.	1. School Health Personnel Questionnaire (SHPQ) Asthma knowledge – 20 items (0–20 points) – higher score, higher knowledge Management and communication – 17 items (5-point Likert scale) (17–85 points) – lower scores indicate better management/ communication skills Confidence – 15 items (7-point Likert scale) (15–105 points) - lower scores indicate better confidence	2
		Asynchronous group (5)			^a^Wimba live classroom - Instructor prerecorded and archived version of the asthma education module.			
		Control group (9)	School nurses in an adjoining county	Received at a different time	Nurses were given information about the study and invited to participate during a working lunch in-service being conducted in their county.			

^a^The training material is not publicly available, and no access was provided by the authors upon request.

### Overall intervention characteristics

The online training programmes varied in delivery and content. Asynchronous formats refer to learning methods where participants learn at their own pace without real-time interaction. These included eBooks^32^, PowerPoint presentation^36^, and web-based training programmes^33–35,37^. In contrast, synchronous formats involve live interactions with instructors and were delivered through platforms such as Google Classroom^38^ and Wimba Live Classroom^39^. Most online training programmes typically lasted for one hour^32,33,36–38^. The content of the interventions commonly covered basic asthma knowledge^32–39^, asthma triggers and symptoms^32–39^, asthma management^32,33,35–39^, and asthma medications^32–39^. One study included broader school-relevant topics such as asthma myths and facts^38^. Some incorporated national guidelines like the Expert Panel Report 3 (EPR-3): Guidelines for the Diagnosis and Management of Asthma and the National Institute of Health (NIH) National Asthma Education Prevention Program (NAEPP) to ensure evidence-based practice^34,39^. Interactive features such as quizzes, videos, and case scenarios were also used to enhance engagement and learning^32–34^.

Out of the eight included training programmes, four were accessible for direct review: Teaming Up for Asthma Control by the University of Missouri^34^, Asthma First Aid for Schools by Asthma Australia^33^, Asthma Basics^37^, and Kickin’ Asthma by the American Lung Association (ALA)^35^, though the latter required a fee. The remaining four online training interventions were not accessible at the time of review and attempts to contact the corresponding authors were unsuccessful^32,36,38,39^. Accessibility was assessed to determine whether the training programmes were still available in their original version, acknowledging that some may have been removed, revised, or updated. If newer versions were identified, they were clearly stated. As the latest versions may differ from those used in the original studies, all data on intervention characteristics and outcomes were extracted solely from the information reported in the published articles.

### Knowledge questionnaire used

Six studies used validated questionnaires to assess asthma knowledge, such as the Asthma First Aid Knowledge Questionnaire (AFAKQ)^32,33^, the Newcastle Asthma Knowledge Questionnaire (NAKQ)^36^, standard tests by the ALA^37^, the Asthma Knowledge Survey^38^, and the School Personnel Questionnaire (SHPQ)^39^. Two studies used a self-developed instrument^34^^,35^. The questionnaires ranged from 11 to 31 items in length and typically included open-ended, true/false, or Likert-scale questions.

### Quality assessment

The overall quality of the studies varied, with scores ranging from 40 to 80%. Luckie et al.^33^ and Nowakowski et al.^37^ achieved high-quality scores of 80%^33,37^. Hacker-Talyor^35^, McAuliff^36^, Nwabuzor^38^, and Putman-Casdorph and Pinto^39^ obtained a moderate score of 60%^35,36,38,39^, while Francis et al.^32^ and Francisco et al.^34^ scored 40%^32,34^ (Table 2).

**Table 2:** Quality assessment of included studies.

Author, year	Quantitative randomised controlled trials					Quantitative non-randomised					Quality score
	2.1	2.2	2.3	2.4	2.5	3.1	3.2	3.3	3.4	3.5	
Francis et al.^32^	Y	C	C	Y	N	-	-	-	-	-	40%
Francisco et al.^34^	-	-	-	-	-	Y	C	N	C	Y	40%
Hacker-Taylor^35^	-	-	-	-	-	N	Y	Y	C	Y	60%
Luckie et al.^33^	-	-	-	-	-	Y	Y	Y	C	Y	80%
McAuliff^36^	-	-	-	-	-	N	Y	Y	C	Y	60%
Nowakowski et al.^37^	-	-	-	-	-	Y	Y	Y	N	Y	80%
Nwabuzor^38^	-	-	-	-	-	N	Y	Y	C	Y	60%
Putman-Casdorph & Pinto^39^	-	-	-	-	-	N	Y	Y	C	Y	60%

The numbers indicate the following: 2.1, is randomisation appropriately performed? 2.2, are the groups comparable at baseline? 2.3, are there complete outcome data? 2.4, are outcome assessors blinded to the intervention provided? 2.5, did the participants adhere to the assigned intervention? 3.1, are the participants representative of the target population? 3.2, are measurements appropriate regarding both the outcome and intervention (or exposure)? 3.3, are there complete outcome data? 3.4, are the confounders accounted for in the design and analysis? 3.5, during the study period, is the intervention administered (or exposure occurred) as intended? Y = yes. N = no. C = can’t tell. Quality scores indicate the percentage of items that meet the quality criteria.

### Summary of outcomes measured

The included studies evaluated the effectiveness of online asthma training programmes for school staff using various outcome measures. Overall, all eight studies^32–39^ demonstrated positive outcomes at level 2 (learning), with two studies^35,37^ also reporting positive outcomes at level 1 (reaction). However, no study extended its evaluation to include observable behaviour change (level 3) or organisational outcomes (level 4).

### Kirkpatrick level 1: reaction

Only two studies^35,37^ evaluated participants’ satisfaction with the asthma training programmes (Table 3). Both reported high levels of satisfaction, with participants perceiving the content as relevant and valuable for managing asthma in school environments. In the Kickin’ Asthma intervention, 56% of participants expressed being very satisfied with the training^35^. Similarly, 83% of participants in the online Asthma 101 programme were very or somewhat satisfied with all aspects of the training^37^. Notably, participants found the section on recognising signs and symptoms of respiratory distress to be the most useful.

**Table 3:** Impact of asthma online training on school staff’s perception and skills.

Author, year	School staff’s perception		Skills	
	High course satisfaction	Improved self-confidence/self-efficacy	Asthma first aid competency	Improved management and communication skills
Francis et al.^32^	-	43.4% increase in mean score from pre-test to post-test	-	-
Francisco et al.^34^	-	-	-	-
Hacker-Taylor^35^	56% were very satisfied with the training	29.5% increase in mean score from pre-test to post-test	-	-
Luckie et al.^33^	-	-	29% were deemed competent in handling a moderate to severe asthma exacerbation	-
McAuliff^36^	-	-	-	-
Nowakowski et al.^37^	83% were very satisfied with the training	-	-	-
Nwabuzor^38^	-	13.4% increase in mean score from pre-test to post-test	-	-
Putman-Casdorph & Pinto^39^	-	48.5% (synchronous) and 26.0% (asynchronous) increase in mean score from pre-test to post-test	-	26.4% (synchronous) and 0.3% (asynchronous) increase in mean score from pre-test to post-test

“-“ indicates that the study did not assess or report results for that category.

### Kirkpatrick level 2: learning

All eight included studies evaluated changes in asthma knowledge by comparing pre- and post-intervention scores using various asthma knowledge questionnaires, including validated tools such as the AFAKQ^32,33^ and the NAKQ^36^. All studies reported improvements in asthma knowledge scores post-intervention (Table 4).

**Table 4:** Impact of asthma online training on school staff’s knowledge.

Author, year	Intervention	Score range (lowest - highest)	Pre-intervention test score (mean)	Post-intervention test score (mean)	Change in mean score	% Change in mean score	*P*-value for change in score (pre vs post)	*P*-value for change in score (between groups)	Effect size (Cohen’s *d*)
**Pre-post studies**									
Francisco et al.^34^	Web-based module	0–100	49.10	90.70	41.60	84.73	<0.001	N/A	SD NR
Hacker-Taylor^35^	Web-based module	0–30	22.58	27.75	5.17	22.90	<0.001	N/A	*1.258
Luckie et al.^33^	Web-based module	0–14	7.90	11.10	3.20	40.51	NR	N/A	SD NR
McAuliff^36^	PowerPoint presentation	0–31	30.30	42.70	12.40	40.92	0.001	N/A	*3.409
^a^Nowakowski et al.^37^	Web-based module	0–100	82.60	87.00	4.40	5.33	NR	N/A	SD NR
Nwabuzor^38^	Google Classroom	0–24	16.71	19.14	2.43	14.54	0.005	N/A	*0.734
**Randomised controlled trials (RCTs)**									
Francis et al.^32^	eBook	0–14	9.78	11.72	1.94	19.84	<0.0001	0.110	*0.779
	Face-to-face training	0–14	9.64	12.04	2.40	24.90	<0.0001		*1.023
**Non-randomised controlled studies**									
Putman-Casdorph & Pinto^39^	Wimba Classroom (Synchronous)	0–20	15.80	16.80	1.00	6.33	NR	No significant difference (*P*-value NR)	**0.537**
	Wimba Classroom (Asynchronous)	0–20	16.80	17.20	0.40	2.38	NR		0.138
	Control	0–20	14.80	15.40	0.60	4.05	NR		**0.246**

Effect sizes are reported using Cohen’s *d*; Bold with asterisk (*) indicates a large effect size (d > 0.6), bold only indicates a medium effect size (d = 0.2–0.6), normal text indicates a small effect size (d < 0.2).

*NR* not reported, *N/A* not applicable, *SD* standard deviation.

^a^The marks are the average percentage of correct responses for each item in the asthma knowledge assessment, not the average mark for participants.

The extent of knowledge improvement following the training varied, with percentage score increases ranging from 2.4^39^ to 84.7%^34^. Similarly, effect sizes ranged from a small effect of *d* = 0.138^39^ to a very large effect of *d* = 3.409^36^, reflecting significant differences in the magnitude of knowledge gains. Two studies that compared the outcomes with a control group reported no significant difference in the knowledge between online training and face-to-face training post-intervention^32,39^.

In addition to improvements in knowledge, several studies also assessed perceived learning outcomes, such as self-confidence and skills in managing asthma (Table 3). Four studies (50%)^32,35,38,39^ reported increases in school staff’s self-confidence or self-efficacy in managing asthma following the intervention. In contrast, practical skills were assessed less frequently. Only one study assessed asthma first aid skills using scenario-based assessments^33^, while another measured asthma management and communication skills through the SHPQ^39^. These assessments, although practical in nature, still fall under level 2 as they reflect demonstrated learning in simulated or self-reported contexts rather than observable behavioural changes in real-life school settings.

## Discussion

This systematic review identified eight studies examining online asthma training programmes for school staff, conducted between 2011 and 2023^32–39^. All included studies were from high-income countries and focused on immediate knowledge gains. The overall quality of the included studies varied, with only two studies rated as high quality^33,37^, which is an important consideration when interpreting the findings. All studies demonstrated significant knowledge improvements in effect size with more than 50% reporting medium to large effect sizes (Cohen’s *d* range: 0.537–3.409). Furthermore, direct comparisons between online and face-to-face formats found no significant difference in learning outcomes^32^^,^^39^. However, none evaluated longer-term behavioural or organisational outcomes, representing a critical evidence gap.

The positive outcomes observed at Kirkpatrick levels 1 and 2 establish a foundation for effective online asthma training^32–39^. The improvement in knowledge scores and high satisfaction ratings indicate that participants found the training relevant and engaging. These findings align with evidence from other fields, such as virtual training in nursing and sexual health education, where participants reported increased knowledge and satisfaction with e-learning content^40,41^. This is particularly important in the school context, where school staff often have limited time for professional development and may feel unprepared to manage students with asthma^8^. The inclusion of self-efficacy measures in some studies further supports the idea that participants not only gained knowledge but also felt more confident in their ability to respond to asthma emergencies^32,35,38,39^. The training programmes covered a wide range of topics, including asthma pathophysiology^37,39^, symptom identification^32–39^, medication use^32–39^, and provision of first aid for asthma^32,33,35–39^, which reflects a comprehensive approach to asthma education. However, the use of scenario-based assessments in several studies suggests that knowledge alone may be insufficient for effective real-world application^32,33^.

A key issue in the current evidence is the variation in how asthma knowledge was assessed. For instance, the NAKQ includes open-ended questions that may be too complex for school staff, potentially limiting its suitability^36^. Several studies used self-developed questionnaires, which were not validated^34,35^. While these tools may be tailored to the training content, the lack of standardisation makes it difficult to compare results across studies and ensure accurate measurement of knowledge change. Developing validated instruments specifically for school staff would allow for more consistent evaluation of training outcomes.

Although knowledge gain is essential, true impact depends on demonstrating safer practices (level 3) and organisational improvements (level 4). The predominance of single-group designs^33–38^ and lack of long-term follow-up^32–36,38,39^ make it impossible to assess real-world applications or school health improvements. Level 3 evaluations are achievable, as demonstrated in other health studies, through methods such as long-term follow-up with supervisor observations, participant self-reports, or workplace audits^40,42,43^. Similarly, level 4 impacts have been documented when public health courses led to institutional policy changes^44^. The lack of level 4 outcomes is concerning, as schools need evidence of organisational benefits to support the continued implementation of training programmes. Potential level 4 outcomes might include reduced asthma-related school absenteeism, fewer hospitalisation and emergency visits, and a decrease in days with interrupted activities due to asthma^45,46^. Face-to-face school asthma programmes were found effective for these outcomes^47^. However, online programme lacks such evidence, and it becomes challenging for schools to justify spending time and resources on these programmes, which may hinder their adoption and sustainability. None of the included studies reported cost-effectiveness comparisons between online and face-to-face training. Such data could help schools make informed decisions, especially given that online training is likely to be more cost-effective and less labour-intensive when implemented on a larger scale. While these assessments require more resources, they provide the most meaningful evidence of training effectiveness^48^.

Online training programmes offer several practical benefits for school settings. Its broad accessibility, cost-effectiveness, standardised content delivery, and the flexibility of self-paced learning make it a feasible option for school staff who often struggle to find time for professional development^49^. The two included studies that directly compared delivery modalities, such as online versus face-to-face, or synchronous versus asynchronous formats, reported no significant difference in learning outcomes^32,39^. Therefore, based on this limited evidence, it can be assumed that online training programmes were not inferior to face-to-face training approaches. However, online formats still fall short in providing the hands-on experience, peer interaction, and real-time feedback that in-person training can offer^22^. This also highlights the importance of considering technological access, such as reliable internet access, availability of digital devices, and adequate digital literacy, when planning online interventions, which could be significant barriers in LMICs^50^. A pragmatic approach is a hybrid model, using online modules for foundational knowledge and refreshers, complemented by targeted in-person sessions to reinforce skills^51^.

In terms of the design of the online interventions, among the four studies using the web-based modules, those that used a combination of videos, voice-over slides, quizzes, and scenario-based learning, showed greater knowledge improvements^33–35^. Francisco’s Teaming Up for Asthma Control (TUAC) programme achieved the highest knowledge improvement (84.73%), likely due to its focused content, alignment with state policy, and contextual relevance to local school needs^34^. In contrast, Nowakowski’s Asthma 101 programme showed small knowledge gain (5.33%)^37^. The programme covered broader foundational topics but reused similar slide content from the in-person training. In addition, participants already had a high level of baseline knowledge, which likely contributed to the limited knowledge gain^37^. This suggests that simply transferring traditional teaching materials into an online format is insufficient without attention to engagement and content relevance^52^.

There are several limitations to this systematic review. First, only eight studies met the inclusion criteria, and all were conducted in the USA and Australia, which limits the generalisability of the findings to other cultural and educational contexts. Secondly, the review included only English-language publications, potentially excluding relevant non-English studies. Thirdly, many studies have a small sample size and lack complete statistical reporting, particularly standard deviations required for calculating effect sizes, and attempts to obtain this information from authors were largely unsuccessful^33,34,37^. Finally, a meta-analysis was not feasible due to significant heterogeneity in study designs, outcome measures, and intervention formats, which led us to adopt a narrative synthesis approach^30^.

### Implications for practice and future research

Despite evidence gaps, consistent knowledge gains across online formats suggested that schools can adopt online asthma training for school staff. A hybrid approach may offer the most practical solution, offering the strengths and limitations of both modalities. Regardless of the delivery method, effective training should align with school policies, emergency plans, and strong partnerships with healthcare providers and families^8,53^.

Future studies should (i) use robust designs (e.g., RCTs), (ii) include 3–6-month follow-up with behavioural measures (level 3) and school-level outcomes (level 4), (iii) employ validated, school-appropriate instruments with standardised reporting tied to Kirkpatrick levels, and (iv) incorporate implementation and economic evaluations to inform scale-up^54^. Current studies often focus only on knowledge retention and programme satisfaction, which may not show real-world effectiveness.

## Conclusion

Online asthma training programmes have proven to improve school staff’s knowledge and satisfaction, and appear to be non-inferior to face-to-face training. Their true effect on student health outcomes remains inconclusive due to lack of evidence on behavioural change or organisational impact. Future research should focus on long-term behavioural and organisational outcomes to bridge the gap between knowledge and practice.

## Supplementary information

Supplementary Material 1

## Data Availability

No datasets were generated or analysed during the current study.
