# Supplementary material for: Effectiveness of online asthma training programmes to improve asthma management among school staff: a systematic review using the Kirkpatrick evaluation model
Source: NPJ Prim Care Respir Med. 2025 Oct 23;35:45. doi: 10.1038/s41533-025-00450-w (PMC12549844; doi:10.1038/s41533-025-00450-w)
Supplement: Supplementary file 1 — Supplementary Material 1 [file 41533_2025_450_MOESM1_ESM.pdf]

Supplementary Material 1

Table 1: PubMed Search Strategies - No. of results: 969 (13/6/2024)

No	Concept	Search Terms	No. of Results
1	**Asthma**	Asthma[tiab] OR<br>Asthmas[tiab] OR<br>“Bronchial Asthma”[tiab] OR<br>Antiasthma[tiab] OR<br>Antiasthmatic[tiab] OR<br>Antiasthmatics[tiab] OR<br>Anti-asthma[tiab] OR<br>Anti-asthmatic[tiab] OR<br>Anti-asthmatics[tiab] OR<br>Wheeze[tiab] OR<br>Wheezes[tiab] OR<br>Wheezing[tiab] OR<br>Bronchospasm[tiab] OR<br>Bronchospasms[tiab] OR<br>Bronchoconstrict[tiab] OR<br>Bronchoconstricts[tiab] OR<br>Bronchoconstriction[tiab] OR<br>Bronchoconstrictions[tiab] OR<br>"Bronchial Hypersensitivity"[tiab:~2] OR<br>"Bronchial Hyperreactivity"[tiab:~2] OR<br>"Respiratory Hypersensitivity"[tiab:~2] OR<br>"Respiratory Hyperreactivity"[tiab:~2] OR<br>Asthma[MeSH] OR<br>Respiratory Sounds[MeSH] OR<br>Bronchial Spasm[MeSH] OR<br>Bronchoconstriction[MeSH] OR<br>Bronchial Hyperreactivity[MeSH] OR<br>Respiratory Hypersensitivity[MeSH]	256,142
2	**Technology**	Online[tiab] OR<br>Internet[tiab] OR<br>Technology[tiab] OR<br>Technologies[tiab] OR<br>Virtual[tiab] OR<br>e-health[tiab] OR<br>ehealth[tiab] OR<br>“Mobile Health”[tiab] OR<br>Digital[tiab] OR<br>Digitals[tiab] OR<br>Digitalization[tiab] OR<br>Digitalisation[tiab] OR<br>Digitalised[tiab] OR<br>Digitalized[tiab] OR<br>e-learning[tiab] OR<br>elearning[tiab] OR<br>Program[tiab] OR	4,486,922

		Programs[tiab] OR<br>Programme[tiab] OR<br>Programmes[tiab] OR<br>Software[tiab] OR<br>Webinar[tiab] OR<br>Webinars[tiab] OR<br>Website[tiab] OR<br>Websites[tiab] OR<br>Web[tiab] OR<br>Webs[tiab] OR<br>Web-based[tiab] OR<br>Application[tiab] OR<br>Applications[tiab] OR<br>“Mobile App”[tiab] OR<br>“Mobile Apps”[tiab] OR<br>Distance[tiab] OR<br>Internet[MeSH] OR<br>Software[MeSH] OR<br>Internet-Based Intervention[MeSH] OR<br>Distance Education[MeSH] OR<br>Computer-Assisted Instruction[MeSH] OR<br>Educational Technology[MeSH] OR<br>Digital Health[MeSH] OR<br>Digital Technology[MeSH] OR<br>Mobile Applications[MeSH] OR<br>Artificial Intelligence[MeSH]	
3	**School**	School OR<br>Schools OR<br>Academy OR<br>Academies OR<br>“Academic Institution” OR<br>“Academic Institutions” OR<br>“Educational Institution” OR<br>“Educational Institutions” OR<br>Schools[MeSH]	8,289,624
4	**Teacher**	Teacher[tiab] OR<br>Teachers[tiab] OR<br>Staff[tiab] OR<br>Staffs[tiab] OR<br>Schoolteacher[tiab] OR<br>Schoolteachers[tiab] OR<br>Educator[tiab] OR<br>Educators[tiab] OR<br>Personnel[tiab] OR<br>Personnels[tiab] OR<br>Nurse[tiab] OR<br>Nurses[tiab] OR<br>Faculty[tiab] OR<br>Faculties[tiab] OR<br>Trainer[tiab] OR	709,887

		Trainers[tiab] OR<br>School Teachers[MeSH] OR<br>Teacher Training[MeSH]	
5		**1 AND 2 AND 3 AND 4**	**969 (13/6/2024)**

Table 2: EBSCO (CINAHL Complete) Search Strategies - No. of results: 398 (13/6/2024)

No	Concept	Search Terms	No. of Results
1	**Asthma**	TI (Asthma OR<br>Asthmas OR<br>“Bronchial Asthma” OR<br>Antiasthma OR<br>Antiasthmatic OR<br>Antiasthmatics OR<br>Anti-asthma OR<br>Anti-asthmatic OR<br>Anti-asthmatics OR<br>Wheeze OR<br>Wheezes OR<br>Wheezing OR<br>Bronchospasm OR<br>Bronchospasms OR<br>Bronchoconstrict OR<br>Bronchoconstricts OR<br>Bronchoconstriction OR<br>Bronchoconstrictions OR<br>(Bronchial N2 Hypersensitivity) OR<br>(Bronchial N2 Hyperreactivity) OR<br>(Respiratory N2 Hypersensitivity) OR<br>(Respiratory N2 Hyperreactivity)) OR<br>AB (Asthma OR<br>Asthmas OR<br>“Bronchial Asthma” OR<br>Antiasthma OR<br>Antiasthmatic OR<br>Antiasthmatics OR<br>Anti-asthma OR<br>Anti-asthmatic OR<br>Anti-asthmatics OR<br>Wheeze OR<br>Wheezes OR<br>Wheezing OR<br>Bronchospasm OR<br>Bronchospasms OR<br>Bronchoconstrict OR<br>Bronchoconstricts OR<br>Bronchoconstriction OR<br>Bronchoconstrictions OR<br>(Bronchial N2 Hypersensitivity) OR<br>(Bronchial N2 Hyperreactivity) OR<br>(Respiratory N2 Hypersensitivity) OR<br>(Respiratory N2 Hyperreactivity)) OR<br>MH (Asthma OR<br>Bronchial Diseases)	52,929

2	**Technology**	TI (Online OR<br>Internet OR<br>Technology OR<br>Technologies OR<br>Virtual OR<br>e-health OR<br>ehealth OR<br>“Mobile Health” OR<br>Digital OR<br>Digitals OR<br>Digitalization OR<br>Digitalisation OR<br>Digitalised OR<br>DigitalizedOR<br>e-learning OR<br>elearning OR<br>Program OR<br>Programs OR<br>Programme OR<br>Programmes OR<br>Software OR<br>Webinar OR<br>Webinars OR<br>Website OR<br>Websites OR<br>Web OR<br>Webs OR<br>Web-based OR<br>Application OR<br>Applications OR<br>“Mobile App” OR<br>“Mobile Apps” OR<br>Distance) OR<br>AB (Online OR<br>Internet OR<br>Technology OR<br>Technologies OR<br>Virtual OR<br>e-health OR<br>ehealth OR<br>“Mobile Health” OR<br>Digital OR<br>Digitals OR<br>Digitalization OR<br>Digitalisation OR<br>Digitalised OR<br>Digitalized OR<br>e-learning OR<br>elearning OR<br>Program OR<br>Programs OR<br>Programme OR<br>Programmes OR<br>Software OR	989,743

		Webinar OR<br>Webinars OR<br>Website OR<br>Websites OR<br>Web OR<br>Webs OR<br>Web-based OR<br>Application OR<br>Applications OR<br>“Mobile App” OR<br>“Mobile Apps” OR<br>Distance) OR<br>MH (Internet-Based Intervention OR<br>Mobile Applications OR<br>Non-Traditional Education OR<br>Computer-Assisted Instruction OR<br>Educational Technology OR<br>Digital Health OR<br>Digital Technology OR<br>Artificial Intelligence)	
3	**School**	School OR<br>Schools OR<br>Academy OR<br>Academies OR<br>“Academic Institution” OR<br>“Academic Institutions” OR<br>“Educational Institution” OR<br>“Educational Institutions” OR<br>MH (Schools)	2,291,069
4	**Teacher**	TI (Teacher OR<br>Teachers OR<br>Staff OR<br>Staffs OR<br>Schoolteacher OR<br>Schoolteachers OR<br>Educator OR<br>Educators OR<br>Personnel OR<br>Personnels OR<br>Nurse OR<br>Nurses OR<br>Faculty OR<br>Faculties OR<br>Trainer OR<br>Trainers OR<br>Employee OR<br>Employees) OR<br>AB (Teacher OR<br>Teachers OR<br>Staff OR	630,737

		Staffs OR<br>Schoolteacher OR<br>Schoolteachers OR<br>Educator OR<br>Educators OR<br>Personnel OR<br>Personnels OR<br>Nurse OR<br>Nurses OR<br>Faculty OR<br>Faculties OR<br>Trainer OR<br>Trainers OR<br>Employee OR<br>Employees) OR<br>MH (Teachers OR<br>Nurses OR<br>Employees OR<br>Faculty)	
5		**1 AND 2 AND 3 AND 4**	**398 (13/6/2024)**

Table 3: Web of Science Search Strategies - No. of results: 942 (13/6/2024)

No	Concept	Search Terms	No. of Results
1	**Asthma**	TS=(Asthma OR<br>Asthmas OR<br>“Bronchial Asthma” OR<br>Antiasthma OR<br>Antiasthmatic OR<br>Antiasthmatics OR<br>Anti-asthma OR<br>Anti-asthmatic OR<br>Anti-asthmatics OR<br>Wheeze OR<br>Wheezes OR<br>Wheezing OR<br>Bronchospasm OR<br>Bronchospasms OR<br>Bronchoconstrict OR<br>Bronchoconstricts OR<br>Bronchoconstriction OR<br>Bronchoconstrictions OR<br>(Bronchial NEAR/2 Hypersensitivity) OR<br>(Bronchial NEAR/2 Hyperreactivity) OR<br>(Respiratory NEAR/2 Hypersensitivity) OR<br>(Respiratory NEAR/2 Hyperreactivity))	246,210
2	**Technology**	TS=(Online OR<br>Internet OR<br>Technology OR<br>Technologies OR<br>Virtual OR<br>e-health OR<br>ehealth OR<br>“Mobile Health” OR<br>Digital OR<br>Digitals OR<br>Digitalization OR<br>Digitalisation OR<br>Digitalised OR<br>Digitalized OR<br>e-learning OR<br>elearning OR<br>Program OR<br>Programs OR<br>Programme OR<br>Programmes OR<br>Software OR<br>Webinar OR<br>Webinars OR<br>Website OR<br>Websites OR	13,066,960

		Web OR<br>Webs OR<br>Web-based OR<br>Application OR<br>Applications OR<br>“Mobile App” OR<br>“Mobile Apps” OR<br>Distance)	
3	**School**	ALL=(School OR<br>Schools OR<br>Academy OR<br>Academies OR<br>“Academic Institution” OR<br>“Academic Institutions” OR<br>“Educational Institution” OR<br>“Educational Institutions”)	19,293,384
4	**Teacher**	TS=(Teacher OR<br>Teachers OR<br>Staff OR<br>Staffs OR<br>Schoolteacher OR<br>Schoolteachers OR<br>Educator OR<br>Educators OR<br>Personnel OR<br>Personnels OR<br>Nurse OR<br>Nurses OR<br>Faculty OR<br>Faculties OR<br>Trainer OR<br>Trainers OR<br>Employee OR<br>Employees)	1,448,332
5		**1 AND 2 AND 3 AND 4**	**942**<br>**(13/6/2024)**

Table 4: Scopus Search Strategies - No. of results: 1,802 (13/6/2024)

No	Concept	Search Terms	No. of Results
1	**Asthma**	TITLE-ABS-KEY(Asthma OR<br>Asthmas OR<br>“Bronchial Asthma” OR<br>Antiasthma OR<br>Antiasthmatic OR<br>Antiasthmatics OR<br>Anti-asthma OR<br>Anti-asthmatic OR<br>Anti-asthmatics OR<br>Wheeze OR<br>Wheezes OR<br>Wheezing OR<br>Bronchospasm OR<br>Bronchospasms OR<br>Bronchoconstrict OR<br>Bronchoconstricts OR<br>Bronchoconstriction OR<br>Bronchoconstrictions OR<br>(Bronchial Pre/2 Hypersensitivity) OR<br>(Bronchial Pre/2 Hyperreactivity) OR<br>(Respiratory Pre/2 Hypersensitivity) OR<br>(Respiratory Pre/2 Hyperreactivity))	351,090
2	**Technology**	TITLE-ABS-KEY(Online OR<br>Internet OR<br>Technology OR<br>Technologies OR<br>Virtual OR<br>e-health OR<br>ehealth OR<br>“Mobile Health” OR<br>Digital OR<br>Digitals OR<br>Digitalization OR<br>Digitalisation OR<br>Digitalised OR<br>Digitalized OR<br>e-learning OR<br>elearning OR<br>Program OR<br>Programs OR<br>Programme OR<br>Programmes OR<br>Software OR<br>Webinar OR<br>Webinars OR<br>Website OR<br>Websites OR	19,232,309

		Web OR<br>Webs OR<br>Web-based OR<br>Application OR<br>Applications OR<br>“Mobile App” OR<br>“Mobile Apps” OR<br>Distance)	
3	**School**	ALL(School OR<br>Schools OR<br>Academy OR<br>Academies OR<br>“Academic institution” OR<br>“Academic institutions” OR<br>“Educational institution” OR<br>“Educational institutions”)	28,809,127
4	**Teacher**	TITLE-ABS-KEY(Teacher OR<br>Teachers OR<br>Staff OR<br>Staffs OR<br>Schoolteacher OR<br>Schoolteachers OR<br>Educator OR<br>Educators OR<br>Personnel OR<br>Personnels OR<br>Nurse OR<br>Nurses OR<br>Faculty OR<br>Faculties OR<br>Trainer OR<br>Trainers OR<br>Employee OR<br>Employees)	2,605,184
5		**1 AND 2 AND 3 AND 4**	**1,802 (13/6/2024)**

Table 5: ProQuest Search Strategies - No. of results: 239 (13/6/2024)

No	Concept	Search Terms	No. of Results
1	**Asthma**	title(Asthma OR<br>Asthmas OR<br>“Bronchial Asthma” OR<br>Antiasthma OR<br>Antiasthmatic OR<br>Antiasthmatics OR<br>Anti-asthma OR<br>Anti-asthmatic OR<br>Anti-asthmatics OR<br>Wheeze OR<br>Wheezes OR<br>Wheezing OR<br>Bronchospasm OR<br>Bronchospasms OR<br>Bronchoconstrict OR<br>Bronchoconstricts OR<br>Bronchoconstriction OR<br>Bronchoconstrictions OR<br>(Bronchial PRE/2 Hypersensitivity) OR<br>(Bronchial PRE/2 Hyperreactivity) OR<br>(Respiratory PRE/2 Hypersensitivity) OR<br>(Respiratory PRE/2 Hyperreactivity)) OR<br>abstract(Asthma OR<br>Asthmas OR<br>“Bronchial Asthma” OR<br>Antiasthma OR<br>Antiasthmatic OR<br>Antiasthmatics OR<br>Anti-asthma OR<br>Anti-asthmatic OR<br>Anti-asthmatics OR<br>Wheeze OR<br>Wheezes OR<br>Wheezing OR<br>Bronchospasm OR<br>Bronchospasms OR<br>Bronchoconstrict OR<br>Bronchoconstricts OR<br>Bronchoconstriction OR<br>Bronchoconstrictions OR<br>(Bronchial PRE/2 Hypersensitivity) OR<br>(Bronchial PRE/2 Hyperreactivity) OR<br>(Respiratory PRE/2 Hypersensitivity) OR<br>(Respiratory PRE/2 Hyperreactivity))	27,837

2	**Technology**	title(Online OR<br>Internet OR<br>Technology OR<br>Technologies OR<br>Virtual OR<br>e-health OR<br>ehealth OR<br>“Mobile Health” OR<br>Digital OR<br>Digitals OR<br>Digitalization OR<br>Digitalisation OR<br>Digitalised OR<br>Digitalized OR<br>e-learning OR<br>elearning OR<br>Program OR<br>Programs OR<br>Programme OR<br>Programmes OR<br>Software OR<br>Webinar OR<br>Webinars OR<br>Website OR<br>Websites OR<br>Web OR<br>Webs OR<br>Web-based OR<br>Application OR<br>Applications OR<br>“Mobile App” OR<br>“Mobile Apps” OR<br>Distance) OR<br>abstract(Online OR<br>Internet OR<br>Technology OR<br>Technologies OR<br>Virtual OR<br>e-health OR<br>ehealth OR<br>“Mobile Health” OR<br>Digital OR<br>Digitals OR<br>Digitalization OR<br>Digitalisation OR<br>Digitalised OR<br>Digitalized OR<br>e-learning OR<br>elearning OR<br>Program OR<br>Programs OR<br>Programme OR<br>Programmes OR<br>Software OR	4,212,748

		Webinar OR<br>Webinars OR<br>Website OR<br>Websites OR<br>Web OR<br>Webs OR<br>Web-based OR<br>Application OR<br>Applications OR<br>“Mobile App” OR<br>“Mobile Apps” OR<br>Distance)	
3	**School**	School OR<br>Schools OR<br>Academy OR<br>Academies OR<br>“Academic institution” OR<br>“Academic institutions” OR<br>“Educational institution” OR<br>“Educational institutions”	5,472,080
4	**Teacher**	title(Teacher OR<br>Teachers OR<br>Staff OR<br>Staffs OR<br>Schoolteacher OR<br>Schoolteachers OR<br>Educator OR<br>Educators OR<br>Personnel OR<br>Personnels OR<br>Nurse OR<br>Nurses OR<br>Faculty OR<br>Faculties OR<br>Trainer OR<br>Trainers OR<br>Employee OR<br>Employees) OR<br>abstract(Teacher OR<br>Teachers OR<br>Staff OR<br>Staffs OR<br>Schoolteacher OR<br>Schoolteachers OR<br>Educator OR<br>Educators OR<br>Personnel OR<br>Personnels OR<br>Nurse OR<br>Nurses OR	756,457

		Faculty OR<br>Faculties OR<br>Trainer OR<br>Trainers OR<br>Employee OR<br>Employees)	
5		**1 AND 2 AND 3 AND 4**	**239 (13/6/2024)**

Table 6: EBSCO (Education Research Complete) Search Strategies - No. of results: 149 (13/6/2024)

No	Concept	Search Terms	No. of Results
1	**Asthma**	TI (Asthma OR<br>Asthmas OR<br>“Bronchial Asthma” OR<br>Antiasthma OR<br>Antiasthmatic OR<br>Antiasthmatics OR<br>Anti-asthma OR<br>Anti-asthmatic OR<br>Anti-asthmatics OR<br>Wheeze OR<br>Wheezes OR<br>Wheezing OR<br>Bronchospasm OR<br>Bronchospasms OR<br>Bronchoconstrict OR<br>Bronchoconstricts OR<br>Bronchoconstriction OR<br>Bronchoconstrictions OR<br>(Bronchial N2 Hypersensitivity) OR<br>(Bronchial N2 Hyperreactivity) OR<br>(Respiratory N2 Hypersensitivity) OR<br>(Respiratory N2 Hyperreactivity)) OR<br>AB (Asthma OR<br>Asthmas OR<br>“Bronchial Asthma” OR<br>Antiasthma OR<br>Antiasthmatic OR<br>Antiasthmatics OR<br>Anti-asthma OR<br>Anti-asthmatic OR<br>Anti-asthmatics OR<br>Wheeze OR<br>Wheezes OR<br>Wheezing OR<br>Bronchospasm OR<br>Bronchospasms OR<br>Bronchoconstrict OR<br>Bronchoconstricts OR<br>Bronchoconstriction OR<br>Bronchoconstrictions OR<br>(Bronchial N2 Hypersensitivity) OR<br>(Bronchial N2 Hyperreactivity) OR<br>(Respiratory N2 Hypersensitivity) OR<br>(Respiratory N2 Hyperreactivity)) OR<br>MH (Asthma OR<br>Bronchial Diseases)	5,073

2	**Technology**	TI (Online OR<br>Internet OR<br>Technology OR<br>Technologies OR<br>Virtual OR<br>e-health OR<br>ehealth OR<br>“Mobile Health” OR<br>Digital OR<br>Digitals OR<br>Digitalization OR<br>Digitalisation OR<br>Digitalised OR<br>DigitalizedOR<br>e-learning OR<br>elearning OR<br>Program OR<br>Programs OR<br>Programme OR<br>Programmes OR<br>Software OR<br>Webinar OR<br>Webinars OR<br>Website OR<br>Websites OR<br>Web OR<br>Webs OR<br>Web-based OR<br>Application OR<br>Applications OR<br>“Mobile App” OR<br>“Mobile Apps” OR<br>Distance) OR<br>AB (Online OR<br>Internet OR<br>Technology OR<br>Technologies OR<br>Virtual OR<br>e-health OR<br>ehealth OR<br>“Mobile Health” OR<br>Digital OR<br>Digitals OR<br>Digitalization OR<br>Digitalisation OR<br>Digitalised OR<br>Digitalized OR<br>e-learning OR<br>elearning OR<br>Program OR<br>Programs OR<br>Programme OR<br>Programmes OR<br>Software OR	1,029,290

		Webinar OR<br>Webinars OR<br>Website OR<br>Websites OR<br>Web OR<br>Webs OR<br>Web-based OR<br>Application OR<br>Applications OR<br>“Mobile App” OR<br>“Mobile Apps” OR<br>Distance) OR<br>MH (Internet-Based Intervention OR<br>Mobile Applications OR<br>Non-Traditional Education OR<br>Computer-Assisted Instruction OR<br>Educational Technology OR<br>Digital Health OR<br>Digital Technology OR<br>Artificial Intelligence)	
3	**School**	School OR<br>Schools OR<br>Academy OR<br>Academies OR<br>“Academic Institution” OR<br>“Academic Institutions” OR<br>“Educational Institution” OR<br>“Educational Institutions” OR<br>MH (Schools)	2,360,779
4	**Teacher**	TI (Teacher OR<br>Teachers OR<br>Staff OR<br>Staffs OR<br>Schoolteacher OR<br>Schoolteachers OR<br>Educator OR<br>Educators OR<br>Personnel OR<br>Personnels OR<br>Nurse OR<br>Nurses OR<br>Faculty OR<br>Faculties OR<br>Trainer OR<br>Trainers OR<br>Employee OR<br>Employees) OR<br>AB (Teacher OR<br>Teachers OR<br>Staff OR	899,970

		Staffs OR<br>Schoolteacher OR<br>Schoolteachers OR<br>Educator OR<br>Educators OR<br>Personnel OR<br>Personnels OR<br>Nurse OR<br>Nurses OR<br>Faculty OR<br>Faculties OR<br>Trainer OR<br>Trainers OR<br>Employee OR<br>Employees) OR<br>MH (Teachers OR<br>Nurses OR<br>Employees OR<br>Faculty)	
5		**1 AND 2 AND 3 AND 4**	**149 (13/6/2024)**

Summary

Database	No. of results (13/6/2024)
PubMed	**969**
CINAHL	**398**
WoS	**942**
Scopus	**1802**
ProQuest	**239**
ERC	**149**
**Total**	**4499**
